# Self-report questionnaires in eating disorders: do we need to be careful interpreting self-report in conditions with self-perception issues?

**DOI:** 10.1192/j.eurpsy.2022.1486

**Published:** 2022-09-01

**Authors:** C. Evans, C. Paz, J.C. Medina, A. Grau

**Affiliations:** 1 Universidad de Las Américas, Ecuador, Psychology, Aime, France; 2 Universidad de Las Américas, Ecuador, Psychology, Quito, Ecuador; 3 Universitat Oberta de Catalunya, Psychology, Barcelona, Spain; 4 ITA Mental Health, Avenir Unit, Barcelona, Spain

**Keywords:** questionnaires, Psychometrics, clinimetrics, Eating Disorders

## Abstract

**Introduction:**

A major revolution in psychiatry since the late 20th and early 21st Century has sought to put the individual client at the heart of intervention, promoting shared decision making. Increasing use of patient reported outcome measures (PROMs) to evaluate interventions and even steer therapies (“power assisted steering for psychotherapy”, Evans 2012) appears congruent with this. But is caution needed interpreting PROMS where self-perception distortions form a core part of the client’s problem? Eating disorders are a paradigmatic test.

**Objectives:**

To see if PROM scores at initial presentation at services for ED seemed congruent with help-seeking. We report CORE-Outcome scores here.

**Methods:**

Inclusion criteria were a diagnosis of an ED and opting in to treatment. Consecutive new clients at all the centres were approached for participation. Scores distributions were analysed to see if numbers of low scores, “non-clinical range” scores seemed congruent with help-seeking.

**Results:**

18% of the participants who completed the CORE-OM at baseline had a score below the Clinically Significant Change (CSC) cutting point. Though the rate was higher in participants with an Anorexia type I diagnosis (22.6%) than those with other ED diagnoses (15.8%): in the expected direction, the difference was narrowly non-significant (chi-squared = 3.5, d.f. = 1, p = .06). Scores did relate to treatment level.

**Figure:**
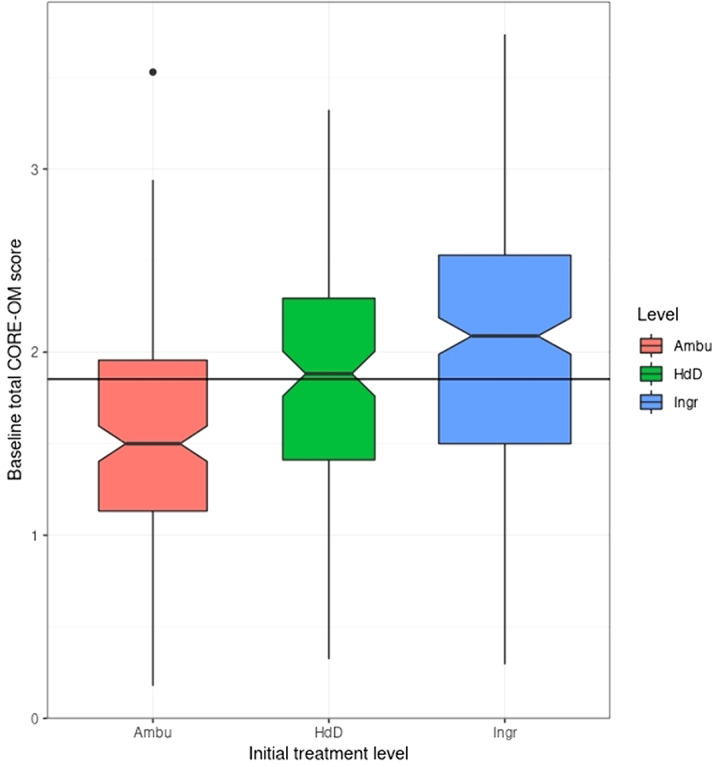

**Conclusions:**

The predicted elevated rate of non-clinical baseline scores in the AN1 group was narrowly non-significant but the rate of 18% non-clinical scores in a help-seeking population raises a cautionary message about interpretation of change from initially low scores.

**Disclosure:**

I am one of the three trustees of CORE System Trust which holds the copyright on the CORE measures used in this study but the measures are all provided under a Creative Commons licence so I receive no remuneration from this.

